# Experiences of Peer Mentoring Sexual and Gender Minority Emerging Adults Who Are at Risk for Suicide: Mixed Methods Study

**DOI:** 10.2196/67814

**Published:** 2025-01-29

**Authors:** Jennifer T Tran, Jessica Webster, James R Wolfe, Jennifer Ben Nathan, Lindiwe Mayinja, Marin Kautz, Maria A Oquendo, Gregory K Brown, David Mandell, Danielle Mowery, José A Bauermeister, Lily A Brown

**Affiliations:** 1 Department of Family and Community Health School of Nursing University of Pennsylvania Philadelphia, PA United States; 2 Department of Psychiatry Perelman School of Medicine University of Pennsylvania Philadelphia, PA United States; 3 Department of Biostatistics Perelman School of Medicine University of Pennsylvania Philadelphia, PA United States

**Keywords:** suicide prevention, peer mentorship, LGBTQIA health, mental health

## Abstract

**Background:**

Sexual and Gender Diverse Youth (SGDY) are at increased risk for suicide due to unique experiences including discrimination, family or friend rejection, and low positive affect. Peer mentors (PMs) may offer a unique opportunity for intervention but are underutilized for suicide prevention among SGDY.

**Objective:**

Little is known about the training needed for PMs when working with SGDY at risk for suicide. We developed an intervention, Supporting Transitions to Adulthood and Reducing Suicide (STARS), to improve suicide prevention among SGDY and increase social support, coping, and positive effects. PMs were trained by a licensed clinical therapist and provided a manual. PMs meet virtually for 6 weeks, providing social support, strategies to diminish the impact of discrimination, connection to safe spaces, and reinforcement of intentions to use Safety Plans with mentees.

**Methods:**

To understand PMs’ experiences in their role, including distress, fidelity to the manual, and perceptions of feasibility and acceptability of STARS and mentees’ Safety Plan, we collected survey data from mentees and PMs as well as in-depth interviews with PMs after the completion of the intervention.

**Results:**

As of September 2024, all peer mentees (N=64) have completed the study and all PMs have finished providing sessions for peer mentees. PMs (n=5) reported overall high comfort (8.52) and low distress (1.93) during sessions. All 5 PMs had high fidelity (>90%) to the PM intervention training. All 5 PMs reported high feasibility (17.50), acceptability (20), and appropriateness (20) of the STARS intervention. Mentees (n=27) reported high confidence ratings (3.54) in speaking with their PMs.

**Conclusions:**

Peer mentorship for SGDY who are at risk for suicide was feasible and acceptable by PMs and mentees alike. PMs reported that they felt comfortable and confident during the sessions. Mentees also reported confidence in working with their PMs. Future research should explore the optimal strategies to support PMs and mentees as they engage in suicide prevention work as well as incorporate feedback from the PMs in this study to ensure optimal outcomes.

**Trial Registration:**

ClinicalTrials.gov NCT05018143; https://clinicaltrials.gov/study/NCT05018143

**International Registered Report Identifier (IRRID):**

RR2-10.2196/48177

## Introduction

Emerging adulthood is a critical period of suicide risk for Sexual and Gender Diverse Youth (SGDY) people [1]. A meta-analysis indicated 11%-20% of sexual and gender diverse (SGD) individuals have a lifetime prevalence of suicide attempts compared with 4% of heterosexual adults [2]. Rates of suicide attempts may be higher; however, as these statistics only include SGDY who have self-reported their sexual and gender minority identity and ignore those who have not “come out” and are at even greater risk for suicidal ideation [2]. A meta-analysis of SGDY and emerging adults (12-20 years old) found that bisexual and transgender youth were at the most significant risk of attempting suicide (odds ratios of 4.87 and 5.87, respectively) compared with their cisgender heterosexual peers [3].

Sexually diverse emerging adults (eg, lesbian, gay, bisexual, transgender, and queer/questioning [LGBTQ]) may present with unique individual-level distal risk factors for suicide, such as the age of coming out, violence, exposure to sexual orientation change or conversion efforts, and discrimination, and proximal risk factors for suicide, such as internalized homophobia, expectations of rejection, and level of social support, which all interact to either increase or decrease the risk for suicide depending on the social environment and the individual’s perceived belongingness within said environment [4-7]. Rooted in both the minority stress theory and the interpersonal theory of suicide, previous work has indicated that sexual and gender minority stress for adolescents and emerging adults is associated with suicidal ideation and attempts through perceived burdensomeness and thwarted belongingness [8]. To address feelings of burdensome and thwarted belongingness, increasing a sense of connectedness for SGM emerging adults can be an important protective factor against suicide, even when accounting for experiences of discrimination and victimization [9].

PM may serve as a strategy to provide social support, promote positive affect and feelings of belongingness, and build a connection to the community. Given at-risk SGM individuals’ tendency to disclose suicidal ideation to peers rather than professionals, peers are crucial actors in suicide prevention [10,11]. Previous research demonstrated that most individuals contemplating suicide, particularly adolescents and young adults, do not seek help from formal support structures due to stigma concerns, but instead use informal resources like friends and family [12]. Therefore, educating peers about suicide, depression, and the resources available for at-risk individuals may reduce their reluctance to intervene and increase their ability to do so appropriately.

A review shows that peer mentoring can effectively promote health behavior changes in adolescents and emerging adults through reinforcing coping skills, incorporating skill-building activities, and delivering social support [13]. In studies of suicide prevention models, peer mentorship decreased stigma related to help-seeking behaviors and reduced rates of repeated psychiatric hospitalization [14,15]. A pilot trial of a peer-delivered safety planning intervention (SPI) found that adults who received the peer-delivered version had fewer emergency room visits during the follow-up period than those who received the provider-delivered version [16]. Those with sexual minority identities reported reluctance to access mental health services for fear of discrimination and dismissal of their emotional distress [17,18]. Therefore, approaches using peer mentorship that decrease stigma and decrease the need for repeated service engagement may be critical for reducing suicide risk in the SGM community.

For SGDY, effective peer mentor (PM) interventions should incorporate several design characteristics to prevent future suicide risk. First, they should target social support [19]. Second, they should involve skill-based peer-administered interventions instead of purely supportive interventions [20,21]. Third, they should market interventions as LBGTQ (lesbian, gay, bisexual, transgender, and queer/questioning)-affirming [22]. Fourth, they should emphasize the use of a safety plan, which reduces the risk of suicide attempts [23]. When SGM emerging adults are reluctant to use their safety plan, peer mentorship should be enhanced with principles of motivational interviewing (MI). MI is a counseling method designed to elicit behavior change [24-27]. MI can be successfully used in peer-based interventions [28-30]. Given the need for peer mentor interventions to consider a multitude of important design characteristics and targets, there is a need to understand the challenges for PMs. Research to understand the experiences of PMs who deliver suicide prevention interventions to SGDY would be instructive.

In this paper, we describe the protocol for training PMs to fidelity in a suicide prevention intervention (Supporting Transitions to Adulthood and Reducing Suicide [STARS]) and detail their experiences in delivering MI- and cognitive behavioral therapy (CBT)–based content to SGDY mentees. We hypothesized that PMs would retain high fidelity to the protocol throughout their participation in the project, operationalized as greater than 80% fidelity to critical components of each session. We report on peer mentor distress and comfort ratings (completed after each peer mentor session) and evaluate changes in distress and comfort throughout the trial. We hypothesized that as PMs gained more experience with the protocol and the population, distress ratings would significantly decrease over time and comfort ratings would increase over time. Finally, we completed qualitative interviews with PMs about their experiences serving in this role. We hypothesized that PMs would report that the STARS intervention was acceptable, feasible, and appropriate.

## Methods

### The STARS Intervention

This study was part of a larger university randomized control trial (NCT05018143) evaluating an app-based intervention STARS aimed to reduce suicidal ideation and behaviors among SGDY (ages 18-24 years) [31]. Eligible participants were between the ages of 18 and 24 years, lived in the Philadelphia Metro area, reported no psychotic symptoms (hallucinations or delusions), had access to a smartphone and Wi-Fi, and had active suicidal ideation in the past month. Participants were randomized to receive an in-person brief, evidence-based SPI (control arm) or to receive the SPI plus access to STARS (intervention arm). Mentees randomized to the STARS intervention had access to a mobile app focused on the provision of life skills and their safety plan and 6 peer mentor sessions. More detailed information on the more extensive study can be found in the study by Brown et al [31].

### PM Training Plan

PMs completed eight 2-hour training sessions with 2 licensed mental health providers. The first session included an overall introduction to MI. PMs received training in core MI concepts, with an emphasis on skill use. We provided a 2-hour training covering ambivalence, MI spirit (ie, acceptance, partnership, compassion, and evocation), and the righting reflex (the inclination helpers have to give advice, correct what they see as wrong information, or the wrong reasoning, and generally fix things for the person they are helping). OARS (ie, open-ended questions, affirmations, reflections, and summaries) skills were covered in a second 2-hour training. A third 2-hour training covered the 4 MI processes (ie, engaging, focusing, evoking, and planning) and the Helping Roadmap model [32]. PMs were assigned exercises after each training session to support their acquisition of skills. These trainings were adapted from materials used in the iReach trial [28] and were led by a masters-prepared research staff member (project manager and research coordinator) with a background in mental health counseling and delivering MI training for PMs.

After training and practice in the style of MI, PMs received 62-hour training sessions on the content of PM sessions. These trainings included a didactic overview with questions and answers, modeled roleplay, and then an observed experiential role-play between the PMs with feedback from the study clinicians. After training in the MI-style and CBT content of the peer mentor intervention, additional time was dedicated to navigating difficult scenarios, including exacerbations in suicide risk, risky behaviors in general, boundary crossings, and building rapport and connection (or repairing ruptures in the peer mentor relationship).

After the original training, PMs attended a weekly group consultation meeting that was attended by 1 to 3 study clinicians. In the weekly meeting, PMs provided an update on the mentees to whom they were assigned, discussed difficulties in the session, and provided examples of successes in the PM relationship. PMs provided feedback and ideas to each other, and the study clinicians provided validation, coaching, and support around content areas that PMs indicated a need for more support (ie, what to do in a crisis and how to engage participants).

Approximately 6 months into the start of study recruitment, the team dedicated two 30-minute supervision sessions to reviewing and refreshing PMs’ MI style.

### Participant Measures: Mentee Feedback

In their follow-up survey (at 2 months), mentees were asked questions about their experience with PM sessions, including: “PM sessions were offered at times that worked for my schedule,” and “I feel confident talking to a STARS PM to discuss what’s going on in my life.” Questions were answered on a Likert scale from 1 (strongly disagree) to 4 (strongly agree).

### PM Measures: PM Comfort and Distress

At the end of each PM session, PMs were asked to fill out a post-session form that assessed issues that arose and distress and comfort during the PM sessions. The PM comfort rating included two questions: (1) “On a scale from 1-10 how comfortable did you feel during this PM session?” (“10” is “very comfortable” and “1” is “the most uncomfortable”) and (2) “Please rate the highest level of distress you experienced during this session on a scale of 1-10.” (“10” is “very distressed” and “1” is “not at all distressed”). Scores on the second question were reverse coded, and the scores for both questions were averaged for a total comfort score ranging from 1 to 10, with higher scores indicating higher comfort.

### Implementation Measures

#### Overview

To determine the extent to which the STARS PM intervention was acceptable, appropriate, and feasible, we adapted the Acceptability of Intervention Measure (AIM), Intervention Appropriateness Measure (IAM), and Feasibility of Intervention Measure (FIM) [33]. The AIM, IAM, and FIM demonstrated strong psychometric properties in a series of 3 studies conducted by Weiner et al [33]. The AIM, IAM, and FIM are 4-item measures asking participants to answer questions on a scale of “1” (“completely disagree”) to “5” (“completely agree”). We adapted the scales for the STARS PM intervention; for example, 1 item from the AIM states, “The STARS peer mentoring intervention meets my approval.”

#### PM Qualitative Interview

Semistructured interviews were conducted with all PMs to gain a deeper understanding of PM experiences and views on the STARS PM intervention. A member of the research team, outside of those providing training or supervising the implementation of the peer mentoring intervention, conducted the interviews with PMs virtually. The interviews lasted about 60 minutes and were audio recorded and transcribed verbatim.

We used a Template Analysis approach, a form of thematic analysis emphasizing hierarchical coding [34-36]. An a priori codebook was created informed by the interview guide. Out of 3 researchers (JT, TB, and JW) coded the first transcript using the codebook as a guide and template. After the first transcript, a consensus was reached, and the codebook was edited with final details. The remaining transcripts were divided between 2 researchers and individually coded [34-36].

### Fidelity Monitoring

PM fidelity to the intervention was assessed using a checklist that evaluated their adherence to content and style [31]. Content was assessed based on whether PMs covered key elements of the session (eg, agenda setting for the day’s session, checking in about safety plan use since the last session, and teaching the designated CBT skill for the session). Possible total content scores for the sessions ranged from 16 to 29, based on how many elements were meant to be covered in each session. The style was assessed based on the PMs’ use of MI skills OARS [25] to facilitate participant learning and engagement with the session content. The style was scored on a scale of 0-5 for whether PMs asked open-ended questions, used affirming statements, used reflections, and used summaries during the session.

PMs’ fidelity to the intervention was assessed across all sessions for the first 2 mentees they saw, allowing for early detection of challenges to fidelity and corrective supervision. After completing the intervention with their first 2 mentees, PMs were assessed randomly for 1 in every 6 sessions per mentee. Sessions were randomly selected for fidelity assessment using a randomized list generator. Out of 2 research team members were tasked with completing fidelity ratings, 10% of which were scored twice to assess interrater reliability.

### Ethical Considerations

The study was reviewed by the university’s institutional review board (protocol # 849500) and was deemed a human-subjects study. Informed consent was obtained from participants in person during the baseline visit. Participants were informed of all the risks and benefits of the study. All information is dedeidentified and all personal information collected is kept private and confidential. Participants received up to US $170 (US $50 for baseline, US $30 for 2 months, US $40 for 4 months, and US $50 for 6 months) through physical or electronic ClinCard.

## Results

### Mentee Description

As of September 2024, all peer mentees (N=64) have completed the study. A total of 32 mentees were randomized to receive the STARS app intervention. One mentee opted not to participate in the PM sessions. Out of 4 mentees did not complete all 6 recommended PM sessions. Of the 4 mentees who did not complete all 6 sessions, 2 only completed PM session 1, 1 completed 4 PM sessions, and 1 completed 5 PM sessions. A total of 27 mentees completed all 6 PM sessions.

#### PM Description

PMs were recruited from the Greater Philadelphia, Pennsylvania area, through job announcements through area university student employment boards, university bulletin boards, and network connections. Applicants were interviewed based on previous education, experience with mentoring, mental health, and LGBTQ advocacy. PMs selected needed to have some previous experience in mentoring; however, other aspects were not required as we intentionally wanted a heterogeneity of PMs in educational or professional experiences parallel to participants in the study. Although we never asked specific questions of applicants or employees, given human resources guidelines, we can provide descriptive information about the 5 PMs who delivered sessions, which we obtained from working closely with them. At hiring, PMs were between the ages of 22 to 26, self-identified as a part of a sexual and gender minority community orally, and over half identified as a racial and ethnic minority.

Out of 5 PMs delivered sessions after completing their training. PMs were assigned to study mentees who were randomized to the STARS intervention. PMs worked with no more than 3 STARS mentees at a time. STARS participants were assigned to PMs based on a list and caseload to prevent overburdening PMs.

#### Fidelity to PM Training

PM fidelity checks were completed by 2 researchers (LB and JW), both trained in therapeutic peer mentorship. PM fidelity to content was calculated for each of the 6 peer mentor sessions. Fidelity percentages were high, ranging from 95.7% to 100%. PM fidelity to style was calculated for each of the 6 PM sessions and was high, ranging from 92.1% to 97.4%.

#### Mentee Feedback on Intervention

STARS mentees provided ratings for PM sessions at 2 months. Mentees (N=28) rated their confidence in talking with a PM as 3.54 (SD 0.64) out of a possible confidence rating of 4.0. Mentees agreed that sessions were offered at times that worked for their schedule as 3.74 (SD 0.53) out of a score of 4.0.

### PM Outcomes

#### PM Implementation Outcomes

PMs (n=5) rated the sessions with a feasibility score (FIM) of 16.40 (SD 3.21), an appropriateness score (IAM) of 18.40 (SD 3.58), and an acceptability score (AIM) of 18.40 (SD 3.58) with a possible maximum score of 20 for all implementation outcomes (Table 1).

**Table 1:** Average fidelity ratings to the training of 6 peer mentor (PM) sessions of a mixed methods study on suicide prevention in sexual and gender-diverse youth.

Measure		Session Time Point						
			1	2	3	4	5	6
**Content total scores**								
	Mean (SD)		15.21 (0.70	20.93 (1.49)	20.33 (1.11)	21.38 (1.76)	17.54 (1.13)	26.71 (0.73)
	Maximum		16	22	21	23	18	29
**Style total scores**								
	Mean (SD)		20 (0)	19.80 (0.77)	19.87 (0.35)	19.92 (0.28)	19.77 (0.60)	19.14 (1.83)
	Maximum		20	20	20	20	20	20

#### PM Comfort and Distress

PMs indicated high comfort ratings after each session and low distress. Paired samples *t* tests indicated that there were no significant differences in comfort (*P*=.44) or distress ratings (*P*=.70) from session 1 to session 6 (Table 2).

**Table 2:** Average comfort and distress ratings self-reported by peer mentors (PMs) for each of the 6 PM sessions in a mixed methods study on a suicide prevention intervention for sexual and gender-diverse youth.

Measure			Session time point					
		1		2	3	4	5	6
**Comfort ratings**								
	N	31		29	29	29	28	27
	Mean (SD)	8.81 (0.79)		8.45 (1.33)	8.38 (1.40)	8.41 (1.18)	8.89 (1.07)	8.52 (1.60)
**Distress ratings**								
	N	31		29	29	29	28	27
	Mean (SD)	1.81 (1.22)		2.52 (1.79)	2.59 (2.15)	2 (0.85)	1.96 (1.04)	1.93 (0.87)

#### PM Experiences

Thematic analysis of the interviews with PMs categorized their experiences into 6 themes: decision for peer mentoring, training experiences, use of the pm manual, interpersonal relationship with mentee, internal struggles and growth, and STARS App Thoughts.

#### Decisions for Peer Mentoring

This theme represents background information on the PMs and their motivations for becoming involved in STARS. PMs worked with STARS for 8 months to 18 months. All PMs indicated they were interested in the role due to their career goals related to mental health, LGBTQ+ research, or clinical work. Many PMs reported wanting to give back or support the LGBTQ+ community, due to their connection to the community. One PM stated, “I was really interested in the study itself. I thought it just sounded like a really cool and meaningful idea that would definitely be helpful for the participants.” While another PM said “reason is my self-identification as a cisgender gay man, and that aligns with my value to get back to the community to support all of them.”

#### Training Experiences

This theme represented information on PMs’ experiences of PM training and how prepared they felt to provide PM sessions to mentees for STARS. All PMs reported that their training was helpful, specifically the role-playing sessions. One PM stated, “I felt like I was very prepared to do everything in relation to the like discussing safety plans and sort of working to brainstorm a safety plan that wasn’t quite working for somebody that I felt like we did so much kind of prep work on, and that was always one of that was like one of my favorite parts, always.” Another PM said, “I think doing the mock sessions was definitely helpful. Where I would be pretending to be the participant in one, and then [another PM] would do the opposite for me. I think that was definitely really helpful.” Some PMs spoke about the importance of having weekly meetings (3/5 PMs), particularly having that space to talk to supervisors and other PMs about their experiences and troubleshooting. A PM stated, “It was nice to have, you know, the weekly meeting and be able to share difficult things that would come up.” One PM indicated a possibility for more practice during training periods. Several PMs noted specific skills or topics they needed clarification on, such as engagement with their STARS mentees. All PMs noted that the continued training (MI) was helpful.

#### Use of PM Manual

PMs were asked to describe their experiences delivering the STARS sessions to trial mentees. This theme includes feedback on barriers, facilitators, and suggestions for improvement in future STARS manualized session content implementation. Overall, PMs described having “good experiences” delivering the session content. Out of 2 noted session lengths varied, attributed to content and the interactions with individual mentees. One PM explains, “I think that some of the sessions felt a little bit more like jam-packed with content than others, but that also depended on the person that you were with, and some people really had nothing to say-- and so you could get through it pretty easily. And with some people that was harder.” One PM expressed concern that mentees “zoned out” when delivering content where they had to “talk too much” per the session script. Out of 3 PMs described feeling “more comfortable” delivering the session scripts with each new mentee experience. For most PMs, the main barrier to delivering session content was covering session content within the 30-minute timeframe. Out of 3 PMs described an imbalance in the length of content from session to session as a barrier. One explained, “Delivering the content. Yeah, that would be one thing that I feel like I usually take a long time in delivering session 5 or 6, and I find it hard to squeeze the time to like 25 or 30. So that can be one thing.”

Out of 3 PMs identified the script in the manual as a primary facilitator to content delivery, describing it as “the way I guided people” and “easy to follow along” during sessions. Another explained, “How it was broken up I feel like it made sense. There was like a good flow to it. And it was nice to have examples of how to phrase things, or how to talk about something or questions to ask. Yeah. So that was helpful.” PMs identified flexibility in content and session structure as potential areas for improving STARS implementation. One PM suggested differentiating between essential and optional session content to help adhere to the time limit of sessions. Similarly, another expressed that increasing content flexibility would allow PMs to make sessions more relevant for individual STARS mentees. One PM noted that “if there were things that I felt like we couldn’t just breeze over or just wasn’t in line with where the script was headed. I think things like that made it hard for me to make sure I was getting all the fidelity items done.”

#### Interpersonal Relationship With Mentees

This theme described any interpersonal challenges experienced in the mentoring role as well as positive changes and growths that they observed in STARS mentees. Some challenges described by PMs included difficulties with engagement, challenges related to participant personality or behavior, missing appointments, texting during sessions, trouble relating to session content, and not using the safety plan. Of the 3 PMs had experiences where they felt mentees were not engaging with session content. One PM explains, “Some of the mentees who I felt weren’t that engaged would be examples that they brought up…And I feel like some mentees would like, give, just like, very like surface-level, or things that weren’t significant or gonna help them. I don’t know if they were avoiding getting deep with me. And, you know, I didn’t like that we couldn’t kinda get beyond that.”

Mentee personalities and behaviors were sometimes a challenge for PMs. Some PMs found it difficult to balance covering the session content while giving space for sharing with more talkative STARS mentees. During the sessions, a PM described challenges with STARS mentees doing other things on their computers and phones. Out of 2 PMs described challenges with STARS mentees who frequently missed appointments and needed to reschedule. A PM stated, “There were some who were like chronically late, or would forget, needed to reschedule.” Resolutions included sending reminders before sessions, changing times, and discussing barriers to scheduling.

While there were some interpersonal challenges, PMs also described growth in their STARS mentees. Out of 2 PMs identified an increase in openness to sharing experiences as an area of growth among STARS mentees. One PM described an example in which a mentee was initially resistant to being on camera at the first meeting due to negative body image and concerns of being seen by others but eventually engaged fully in video sessions as the relationship developed, indicating a relationship of safety and trust. All 5 PMs identified a willingness to practice and apply session content outside of weekly meetings as a primary growth area among STARS mentees. One PM noted, “The participants, mostly all of them I had like they were actually implementing [session content]. Like all of the participants, that kind of surprised me a bit. “Cause they actually were using the things that [STARS] was wanting to do. Which is the goal. Yeah. It was cool.” In one example, a PM noticed that STARS mentees who gave positive session feedback shared that they were also using the app and practicing content between sessions. Out of 2 PMs described a noticeable shift in mentees’ motivation and engagement around setting and following through on achieving goals. One PM explained, “I mean, a lot of people were reporting, I think, less suicidal ideation or more ease in using a safety plan and more sort of like feeling like they were able to ride the waves of something which was great and hmm. Yeah, I think just a lot of people developed coping skills.”

#### Internal Struggles and Growth

This theme represented information on the STARS PMs’ internal experiences throughout the study, including emotional responses and reactions to their interactions with mentees, both positive and negative. When discussing concerns about providing peer mentorship to mentees with a history of suicide risk, all 5 PMs mentioned a fear that a mentee would actively be in crisis during a session. They were comforted knowing that they had a script and protocol to follow if something did happen. One PM noted, “One major concern was just like how to go about like if they feel like they’re in crisis while they’re with me. And then another one was how to respond if someone says they want to harm themselves. I remember learning during the sessions.” Despite the safeguards in place, the potential for suicidal crises remained a source of anxiety. Some other internal, emotional, or personal challenges that PMs encountered included worries about their STARS mentees’ health, difficulty hearing about mentees’ history with suicidal ideation, and not being sure if they were making sense or being engaging to their STARS mentees. One person said that these challenges subsided the more “exposure” they had. Another PM felt frustrated by needing to balance helping the STARS mentee with sticking to the script to keep their fidelity rating high.

While PMs expressed some internal struggles, they managed difficult emotions that came up by mentioning them to the principal investigators during weekly team meetings and referring to the script in moments of uncertainty. Out of 2 PMs mentioned the comfort that the script brought them. A PM stated, “I'm sticking to the scripts, and hitting all the items I need to hit… When those goals would kind of work against what I think is most important, to really just see what is most useful for the participant and see what I could do to help them.” Out of 2 other PMs said it was helpful to remind themselves of the boundaries of this role and that the STARS mentees’ lives outside of PM sessions were not in their control.

PMs also indicated that the experience was rewarding seeing some improvement in their STARS mentees. This ranged from simply feeling helpful and effective in their sessions to feeling like they were imparting something useful to seeing STARS mentees use their safety plan between sessions. A PM stated, “I think it was just like rewarding to be able to feel like I’m imparting something useful to the peer mentees. So it was nice when we would have like, you know, a fruitful discussion that felt like it was something that was helpful. And that they could use in their real life and use to make changes that were helpful to them.” One PM felt rewarded by getting encouraging supervisor feedback and hearing STARS mentees express gratitude. In addition, another PM appreciated the opportunity to interact more with the queer community and learn about experiences different from their own.

## Discussion

### Principal Findings

This study aimed to evaluate the experiences of PMs who deliver suicide prevention content to SGDY. The study was designed to provide PMs with comprehensive training for working with SGDY at risk for suicide, evaluate PM experiences (reported ratings of comfort and distress), and assess PM fidelity to the protocol (PM training).

It has been well-documented that there is anxiety in providing care for patients at risk for suicide in behavioral health practitioners [37]. Therefore, it is important to assess comfortability and distress with PMs working in suicide prevention interventions. Overall, PMs described high comfort and low distress ratings after each PM session. The comfort and distress ratings did not change over time. These ratings were aligned with the reported experiences of PMs during the follow-up interviews. PMs described having “good experiences” in delivering the session content and several reported that they felt “more comfortable” as they saw more mentees. Many PMs attributed their comfort with sessions by mentioning them during weekly team meetings. Some PMs stated that having the manual was a comfort during the sessions as a resource for them to refer. Therefore, having regular supervision and a thorough resource (such as a manual) are important components to include in future PM based interventions for SGDY at risk for suicide. To our knowledge, this is the first study to report on PM comfort and distress levels when providing support to mentees in a suicide prevention intervention. These results highlight the importance of structured supervision for PMs to aid in providing support to mentees at risk of suicide knowing the anxiety that can surround such a stigmatizing topic [37].

In examining the fidelity of PM sessions with the training, PMs had high fidelity overall (greater than 90% in content and style fidelity ratings). Fidelity (the degree to which an intervention, ie, peer mentoring, was delivered as intended) [38] plays an important role in how well an intervention is considered successful. Fidelity to an intervention provides information that can help determine if the intervention was delivered as intended and therefore any impacts can be more confidently associated with the intervention. PMs provided sessions to STARS mentees with high fidelity; however, PMs noted some challenges. PMs were made aware of fidelity to the training and session content were being assessed and expressed concerns about completing all the fidelity items in the allotted time limit (30-minute sessions). PMs noted that there was a lot of content to cover during the sessions, but they also wanted to provide space and time for their STARS mentees to share updates. PMs expressed frustration with sticking to the script and addressing all the fidelity content items while also preserving fidelity style. However, we did not interview or collect data pertaining to the STARS mentees experience with their PMs and cannot confirm if this was a similar experience for STARS mentees. These concerns from PMs have been noted by mental health professionals who advocate for the use of a manual in treatment [39]. One noted suggestion is the idea of flexibility in fidelity which refers to the implementation of an intervention protocol that contains the core attributes for fidelity but also allows for flexibility for each individual client [40]. A suggestion for future implementation should include highlighting content that is necessary. Determining what content is necessary can come from feedback provided by licensed mental health professionals, PMs, and mentees. Future iterations of the intervention should include flexibility in fidelity [40] within the manual and training by reducing content and increasing space for PMs and STARS mentees to connect for social support.

The STARS PM intervention was developed with several components for a culturally informed design including (1) targeting social support, (2) skill-based training for PMs, (3) focusing on SGM-affirming context, and (4) highlighting safety plan use [19-23]. In our interviews with PMs, we noted how PMs experience delivering the PM intervention to STARS mentees and how they align with the 4 factors incorporated into the intervention design. For the first factor of social support, PMs reported that mentees expressed gratitude and that they noticed improvement in their mentees. However, as mentioned above, we did not directly collect data from STARS mentees to determine if they felt supported by their PM. Future iterations of the study should look to collected data from STARS mentees and their experiences. Second, PMs highlighted the importance and helpfulness of the skill-based training that was provided, with MI and role-playing. All PMs noted that initial and continual training in MI skills were helpful in conducting sessions with STARS mentees. Some even noted even more training in MI would be helpful. Third, PMs all noted that they wanted to be mentors because they wanted to provide and support the SGD community. While we did not assess whether STARS mentees felt affirmed, we can note that the content delivered and provided for were SGD-affirming. Finally, the fourth component of the safety plan use was reiterated within the training and fidelity of the PMs’ sessions with STARS mentees. During all sessions, PMs were asked to check in with STARS mentees and their use of the safety plan. Some PMs noted the difficulty in how to engage STARS mentees with the safety plan, particularly when mentees noted that they had not looked at or used their safety plan.

Our study provides needed context and implementation information regarding peer-based interventions for suicide prevention for SGDY. In a recent scoping review, Bowersox et al [41] identified very few peer-based interventions for suicide prevention overall. Researchers identified only 9 peer-based interventions aimed at crisis and relapse prevention: however, none of these focused on SGD communities. In a PM program for older adults, Van Orden et al [42] trained senior companions (55 years and older) on topics of confidentiality, reporting requirements, accommodations for disabilities, and common physical and mental health conditions but not in suicide risk. Another program, PREVAIL (Peers for Valued Living), is a trial for adapting peer support delivered intervention for veterans recently hospitalized for suicidal thoughts or behaviors [43]. In qualitative interviews with key parties (veterans with current or recent suicide risk, suicide prevention coordinator, clinicians, peer specialists, and a director of inpatient psychiatry), researchers noted that peers should be trained in topics specific to suicide prevention as well as more general clinical approaches such as MI. These findings inspired the focus in our project of offering practical skills training for reducing suicide risk using a style of MI. There are possible applications of the mentoring model developed in our study (ie, training structure, supervision support, and MI skills) for other communities at risk of suicide, including veterans and older adults.

### Limitations and Future Directions

Limitations of our study include the small sample of PMs (n=5). The PMs may not have been representative of the whole pool of persons that would be PMs for our study as we were unable to recruit directly stating PMs with lived experiences of suicidal ideation and identify as a part of sexual and gender minority communities. Another limitation of the study is the lack of feedback from mentees on their experiences with PM sessions. Future studies should include gathering qualitative data from mentees on their experiences. Future directions should include simultaneous mixed methods study design of qualitative interviews with PMs and participants to understand the relationship between peers as it relates to quantitative outcomes (ie, thwarted belongingness) of participants identifying as SGM emerging adults.

### Conclusion

PMs are outstanding candidates for delivering suicide prevention services, particularly when working with historically oppressed communities, such as SGDY. In this study, we demonstrated the feasibility and acceptability of training and implementing peer mentorship for SGDY who are at higher risk for suicide. PMs reported high ratings of comfort and low ratings of distress when implementing the STARS sessions. In addition, PMs had high fidelity ratings throughout the intervention. Finally, PMs offered helpful suggestions for improving our STARS app and PM content and style to improve outcomes in the future.

## Abbreviations

AIM: Acceptability of Intervention Measure
CBT: cognitive behavioral therapy
FIM: Feasibility of Intervention Measure
IAM: Intervention Appropriateness Measure
LGBTQ: lesbian, gay, bisexual, transgender, and queer/questioning
MI: motivational interviewing
OARS: open-ended questions, affirmations, reflections, and summaries
PM: peer mentor
PREVAIL: Peers for Valued Living
SGD: sexual and gender diverse
SGDY: sexual and gender diverse youth
SPI: safety planning intervention
STARS: Supporting Transitions to Adulthood and Reducing Suicide

## Data Availability

The datasets generated and analyzed during this study are not publicly available due to the sensitive nature of the data collected but are available from the corresponding author on reasonable request.
